# Assessing Apps for Health Care Workers Using the ISYScore-Pro Scale: Development and Validation Study

**DOI:** 10.2196/17660

**Published:** 2021-07-21

**Authors:** Inmaculada Grau-Corral, Percy Efrain Pantoja, Francisco J Grajales III, Belchin Kostov, Valentín Aragunde, Marta Puig-Soler, Daria Roca, Elvira Couto, Antoni Sisó-Almirall

**Affiliations:** 1 Fundacion iSYS (internet, Salud y Sociedad) Barcelona Spain; 2 Hospital Clínic Barcelona Barcelona Spain; 3 Iberoamerican Cochrane Centre Biomedical Research Institute Sant Pau (IRB Sant Pau) Barcelona Spain; 4 University of British Columbia Vancouver, BC Canada; 5 Primary Healthcare Transversal Research Group Institut d’Investigacions Biomèdiques August Pi i Sunyer (IDIBAPS) Barcelona Spain; 6 Casanova Primary Health-Care Center Consorci d’Atenció Primària de Salut Barcelona Esquerra Barcelona Spain; 7 Universitat de Barcelona Barcelona Spain; 8 Les Corts Primary Health-Care Center Consorci d’Atenció Primària de Salut Barcelona Esquerra Barcelona Spain

**Keywords:** assessment, mobile app, mobile application, mHealth, health care professionals, mobile application rating scale, scale development

## Abstract

**Background:**

The presence of mobile phone and smart devices has allowed for the use of mobile apps to support patient care. However, there is a paucity in our knowledge regarding recommendations for mobile apps specific to health care professionals.

**Objective:**

The aim of this study is to establish a validated instrument to assess mobile apps for health care providers and health systems. Our objective is to create and validate a tool that evaluates mobile health apps aimed at health care professionals based on a trust, utility, and interest scale.

**Methods:**

A five-step methodology framework guided our approach. The first step consisted of building a scale to evaluate apps for health care professionals based on a literature review. This was followed with expert panel validation through a Delphi method of (rated) web-based questionnaires to empirically evaluate the inclusion and weight of the indicators identified through the literature review. Repeated iterations were followed until a consensus greater than 75% was reached. The scale was then tested using a pilot to assess reliability. Interrater agreement of the pilot was measured using a weighted Cohen kappa.

**Results:**

Using a literature review, a first draft of the scale was developed. This was followed with two Delphi rounds between the local research group and an external panel of experts. After consensus was reached, the resulting ISYScore-Pro 17-item scale was tested. A total of 280 apps were originally identified for potential testing (140 iOS apps and 140 Android apps). These were categorized using International Statistical Classification of Diseases, Tenth Revision. Once duplicates were removed and they were downloaded to confirm their specificity to the target audience (ie, health care professionals), 66 remained. Of these, only 18 met the final criteria for inclusion in validating the ISYScore-Pro scale (interrator reliabilty 92.2%; kappa 0.840, 95% CI 0.834-0.847; *P*<.001).

**Conclusions:**

We have developed a reproducible methodology to objectively evaluate mobile health apps targeted to health care professionals and providers, the ISYScore-Pro scale. Future research will be needed to adapt the scale to other languages and across other domains (eg, legal compliance or security).

## Introduction

Information and communication technologies offer countless opportunities for knowledge management. Access, collection, and production of information are redefined by information technology with digitization [1]. In the health sector, where knowledge management is central to every process, the digitization of information has had an enormous impact. Moreover, health professionals are incorporating information technology into nearly every aspect of patient care and research.

As digitization of health care and health services expand, the landscape of digital health innovates, transforms, and scales for mass use and adoption. Dorsey and Topol [2] identified three new linked trends in how the health and medical community’s concept of digital health evolves in this everchanging landscape. From the old paradigm in the concept of digital health—picturing increased accessibility, managing acute conditions, and facilitating communication between hospitals and health providers—towards a new scene where digital health is used for convenience services, management of patients with chronic or episodic disease, and for increased communication with patients, digital health enables the objective control of episodic and chronic conditions, and increased communication between providers and patients through their mobile devices. Mobile apps are a large driving force in improving (digital health) capacity for health systems.

Since the launch of mobile app platforms in 2008, people have had access to apps aimed at personal health management. As the popularity of these platforms has increased, their adoption has expanded. As of March 2021, the health and fitness apps represented 2.98% and those of Medicine, 1.88% [3]. Digital health apps have the potential to continue improving health and medical community concerns such as patient follow-up and monitoring, adherence to treatments, and promotion of healthy habits [4,5]. However, there is a need for a clearer understanding of best practices to evaluate the digital health apps.

Fieldwork has clarified the need for each professional and academic domain to understand and capture the needs of potential users. In the last few years, one of the gaps documented in the field is the need to develop and validate new mobile health (mHealth) assessment tools. New research needs to emerge from multidisciplinary and experienced teams to create convergence and integrate methodologies to improve consistency in the mHealth app market [6,7]. For example, theoretical frameworks like the Technology Acceptance Model (TAM) address some of the needs from behavioral research but fall short in terms of mHealth evaluation methods.

To expand on this, from the behavioral perspective, the TAM [8-11] describes the factors as to why users may uptake new technology. This model proposes that when users are faced with a new technology, perceived utility and ease of use will shape their decisions. From the lens of health care professionals, the TAM suggests that new technologies or apps should provide solutions for or to assist with the needs of the clinical practice. Enabling or enhancing patient care will thus become a top priority, ranging from diagnosis to socialization and during interparty communication.

A wide range of studies have evaluated the use of digital tools to enable or enhance patient care. These include, for example, the use of telemedicine to follow patients with chronic diseases (eg, diabetes [12] and lung cancer [13]), disease-relevant education (eg, lymphedema management [14]), and exercise and physical activity monitoring following a cardiac event for secondary prevention [15], along with the use of virtual communities to improve self-care and patient engagement, such as Forumclinic at Hospital Clínic de Barcelona [16].

Today, there is a great societal need to document how to create great experiences through digital health apps, to evaluate these interventions, and to solve the pain points of both patients and health care professionals. High-quality apps can catalyze the efficient translations of new research findings into daily clinical practice, strengthen knowledge translation from the lab to the bedside, and may even radically transform patient care. In this journey, establishing a validated scale to assess the trust, utility, and interest of mobile apps used by health care professionals is the first step to understand how these tools may impact the quality of care delivered and the outcomes of patients receiving care.

Despite multiple efforts to develop rating scales to evaluate apps used in health care [7,17,18], as of the writing of this paper, not a single one (to our knowledge) has empirically addressed how these tools may support day-to-day clinical practice.

The Internet, Health and Society Foundation (Fundación Internet, Salud y Sociedad [ISYS] in Spanish) works with journal editors and members of professional associations, and collaborates with a diverse group of experts to distil best practices in the digital health domain. In 2014, the ISYS developed a scale to evaluate the quality of mHealth apps for patients (the ISYScore for patients or ISYScore-Pac) [18]. This study builds on this earlier research and expands upon its applicability for health care professionals.

The goal of this study is to develop a tool that evaluates health care apps targeted to health care professionals and medical workers at the bedside. The scale was designed specifically to assess interest, trust, and usefulness, and allow for empirical replicability across these dimensions. The scoring system that supported the development of this scale is also presented.

## Methods

The methodology proposed by Moher [19] in his work on health research reporting guidelines was used for the assessment portion. Limitations from other rating scales were considered [7]. This study involved an iterative sequence of a five-step methodology for the assessment of the scale: (1) investigation group definition, (2) theoretical framework and literature review, (3) scale draft, (4) expert panel definition and Delphi rating, and (5) pilot and first scale.

### Investigation Group Definition

The local investigation group was defined as a local *Fundación ISYS* research team with experience in telemedicine and assessing other scales [20], and with strong knowledge on different domains (1 engineer, 5 medical specialists, 1 nurse, and 1 biostatistician). Profiles and expertise from the local investigation group members are available in Multimedia Appendix 1.

### Theoretical Framework and Literature Review

A comprehensive literature search was conducted by the local investigation group to trace possible backgrounds, to collect a selection of theories that had been used in digital health interventions, and to perform a scope of other scales, focusing on the ones targeted specifically to health professionals. Papers were retrieved from the PubMed database with all articles that include “assessment” AND “healthcare professional” AND “mobile applications” with no language or time limitations.

The local investigation group used the TAM framework [8-11] and the vision of persuasive technology conceptualized by Fogg’s functional triad: information and communication technology that function as tools, media, and social actors [21,22].

### Draft of the Scale

The development, implementation, usability, viability, and acceptance information of existing models were extracted. The local investigation group classified assessment criteria into categories and subcategories, and developed the scale items and descriptors. Considerations for the final scale were the dimension values: trust, utility, and interest (Textbox 1). Iterative corrections were made until consensus was reached.

**The trust, utility, and interest model presented to the expert panel (ISYScore-Pro).**

**Trust**

- A1. Validated by a health agency, scientific society, health care professional college, or nongovernmental organization
- A2. Authors are explicitly identified
- A3. Website is accessible (responsibility)
- A4. Cites peer-reviewed sources
- A5. Names the organization responsible
- A6. Updated within the last calendar year
- A7. Disclosure on how the app was financed

**Utility**

- Technology as a tool (increases capacity)
  B1. Provides calculations and measurements
  B2. Helps in a care procedure
  B3. Archives data images
- Technology as a medium (increases the experience)
  B4. Facilitates observation of cause-effect relationships and allows users to rehearse
  B5. Facilitates observation of those who do well (vicarious learning)
  B6. Facilitates patient follow-up
- Technology as social actor (increases social relationships)
  B7. Obtains positive feedback
  B8. Provides social content

**Interest**

- C1. Positive user ratings/downloads
- C2. Available on two platforms (18 items were selected from the review of the literature; this item was removed after local investigation group and external panel of experts agreement)
- C3. Content available in other formats (eg, web, tablet, or magazines)

The main purpose of this phase’s outcome was to define objective indicators that cover all three dimensions of the assessment scale. *Trust* constitutes a dimension crucial for health apps. It would evaluate the robustness of the relationship that a mobile app has with scientific evidence, the frequency in which its information is updated, and the declaration of possible conflicts of interest. For *utility*, the local investigation group chose indicators inspired by the Fogg triad [21,22]. Within this dimension, technology is evaluated as the capacity to enhance or improve experience or as a social actor. For health professionals, perceived ease of use falls within the user *interest* dimension of the ISYScore-Pro, such as evaluating the availability of the mobile app across different platforms, which is valued by some in the scientific community.

### The External Panel of Experts and Delphi Rating

The external panel of experts was selected considering all backgrounds that can help us to validate the 18-item scale draft (Multimedia Appendix 2). An attempt was made to look for heterogeneity. Doctors of different specialties (cardiology, pneumology, pediatrics, surgery, public health, etc); nurses; psychologists and psychiatrists; and experts in physical education, pharmacy, and technology were recruited.

For the expertise profiles in panel selection, the approach [20] was made looking at Twitter, Facebook, and the local investigation group professional network. A wide range of health care professionals, all of them well-known influencers, key opinion leaders, and users of mobile technology in Spain and recognized as scientists and scientific or evidence-based disseminators on eHealth, were contacted. Due to distance and budget limitations, they were only contacted through email, social media, or other publicly available data. In total, 35 Spanish eHealth experts were invited to be part of the panel.

**Figure 1:**
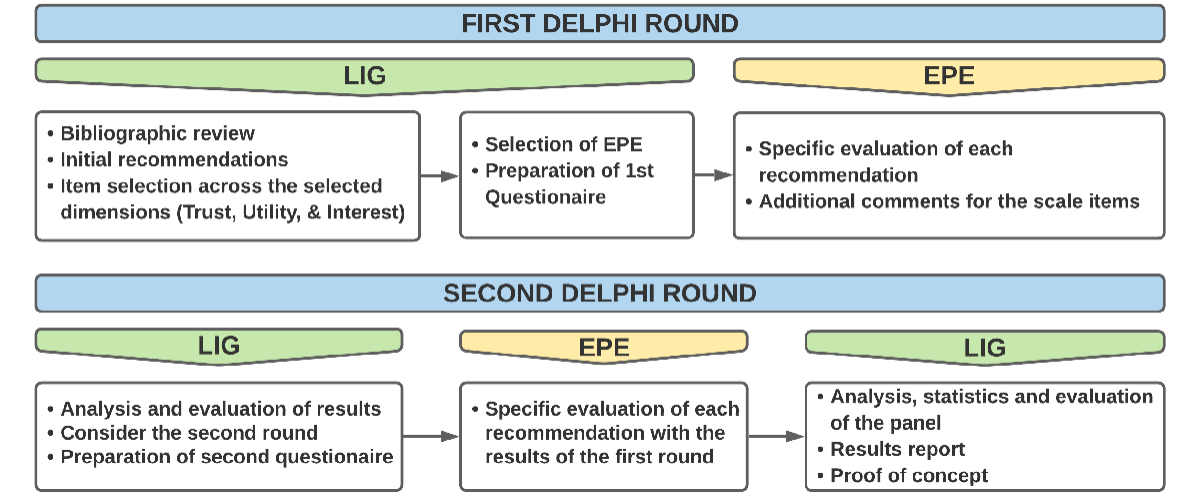
Delphi flowchart. EPE: external panel of experts; LIG: local investigation group.

For feedback on the 18-item scale draft, a Delphi process [18,23,24] was followed. A minimum participation of 70% was needed [6] for each Delphi round. Participant agreement was also set at 75%, as advised by the literature [23-26]. Two rounds of questionnaires were sent out to the external panel of experts. Responses were aggregated and shared with the local investigation group after each round (Figure 1).

The external panel of experts had to assess whether they would include each category in the draft scale (Textbox 1) and, if included, assign a weight to it on a scale from 1 to 5 (0 was used to exclude the category altogether). They were also asked to consider adding a new indicator not proposed in the first round only.

### Pilot and Scale Testing

To test the scale’s performance, we gathered a sample of apps to evaluate. For sampling, the local investigation group used the automated method for capturing apps with Google advanced search tools. For summary purposes, this method explores different results by disease clusters. For clustering, the local investigation group selected keywords from the disease groups in the *International Statistical Classification of Diseases, Tenth Revision* (*ICD-10*) [27]. Pregnancy-related apps and non-disease–related codes where discarded, which brought the total of disease groups explored to 14.

For each *ICD-10* cluster, the first 10 search results on iTunes and the first 10 in Google Play were kept. A total of 280 apps were collected: 140 from Google Play and 140 from iTunes. The strategy in Spanish for each cluster search can be found in Multimedia Appendix 3.

For this pilot, the inclusion criteria included that the app was in the local language (Spanish), the target audience was health care professionals, and its general availability did not require passwords or specific geographies. As exclusion criteria, accuracy was considered, excluding, for example, apps that mention cancer as horoscope. eBooks and podcasts were also excluded. The local investigation group also established that the most recent update had to be within the previous calendar year irrespective of the number of downloads at any time. After duplicates were removed and the inclusion and exclusion criteria were applied, 66 apps remained, and the scale was applied. By consensus the local investigation group established that apps had to meet a minimum cut-off score of no less than 4 items of the final ISYScore-Pro scale.

To evaluate the reliability of the scale the ISYScore-Pro was applied to the final sample of 66 apps. The local investigation group reviewers analyzed apps in pairs. In case of discrepancies, each subgroup discussed the findings and reached a consensus. If consensus was not reached, a third researcher solved the discrepancies [28,29].

Interrater reliability was measured on nominal variables using Cohen kappa with STATA v15.0 (StataCorp) [29]. The target aim was to reach >80% agreement. As the final score includes different categories, a weighted kappa calculation was used. The local investigation group considered the agreement *very low* for kappa results lower than 0.20, *low* for 0.20 to 0.39, *moderate* for 0.40 to 0.59, *high* for 0.60 to 0.79, and *very high or excellent* for 0.80 or higher.

## Results

### Scale Draft

The literature review indicated that existing models do not sufficiently overlap or integrate to provide a framework for rating mobile apps specific to health care professionals. The main mHealth domains were at the individual, organizational, and contextual levels. Existing scales included usability (perceived) and ease of use, design and technology aspects, cost, aesthetics, time, privacy and security, familiarity with technology, risk-benefit assessment, and interaction with others (colleagues, patients, and management) [7,8,30,31]. Based on these findings and the prior work on the ISYScore-Pa scale, our rating scale methodology tested three dimensions (Textbox 1): trust, utility, and interest [8-10,21,32-34].

### Delphi Rating of the Draft Scale and Final Scale

**Figure 2:**
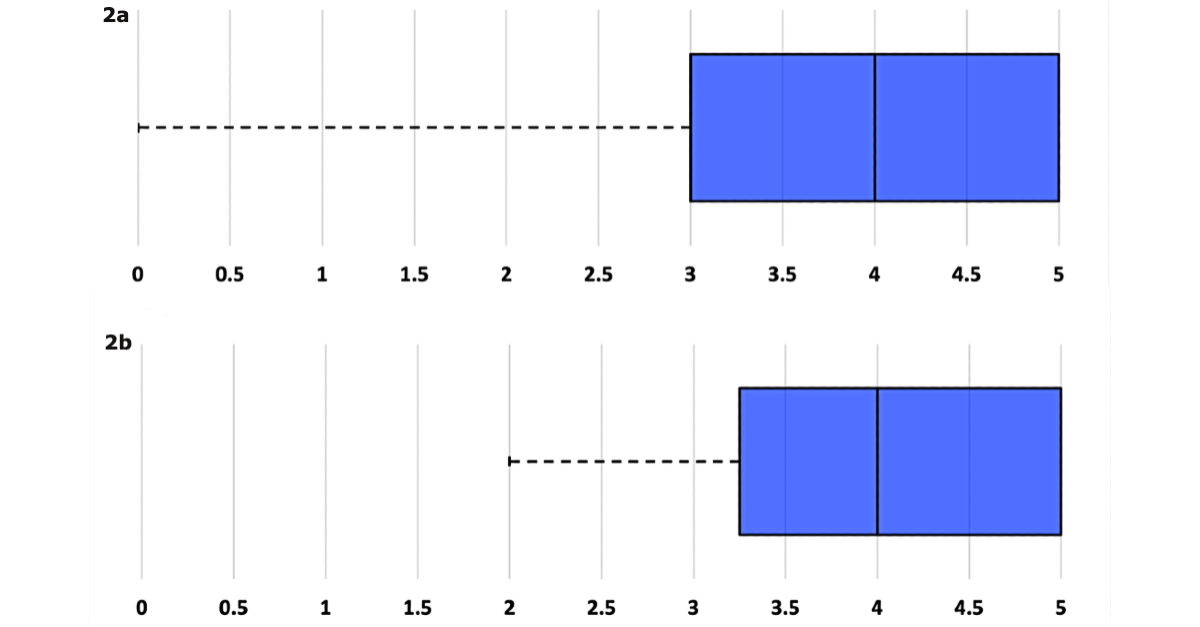
Examples of graphics sent to the external panel of experts (EPE) for the second interaction. After the first interaction, boxplots were sent to the EPE members for a second interaction. (2a) This boxplot example reflects an item with low consensual rate, catalogued by one with a zero value; later on, this item was not included. (2b) This boxplot is an example of an included item, well valuated in the first interaction.

The external panel of experts members completed the study’s two rounds of measure ratings. From the 35 contacted people, 28 (80%) participated in the first round, and 25 (71.4%) in the second one. After the first interaction, results (ie, Figure 2) were sent for a second interaction to the external panel of experts members. From the 18 items originally proposed (Textbox 1), 17 (94.4%) were included in the final draft (C3 was discarded). The Delphi interactions database is available upon request from the corresponding author.

### Scale Reliability and Interrater Agreement

A total of 66 apps were used to test the reliability of the scale. Of these, 13 were excluded, as their score was equal to or lower than 12 (out of 17), which was deemed as the minimum cut-off. The breakdown of the apps evaluated is presented in Table 1. Apps were stratified according to their *ICD-10* disease cluster. Interrater reliability scores were also calculated.

**Table 1:** Evaluation of the agreement between raters, weighted by *ICD-10*^a^ group cluster and app.

*ICD-10* cluster	Apps included for score evaluation		
	Google Play, n	iTunes, n	Total^b^, n
I. Certain infectious and parasitic diseases	2	2	3
II. Neoplasms	3	4	5
III. Diseases of the blood and blood-forming organs and certain disorders involving the immune mechanism	7	2	9
IV. Endocrine, nutritional, and metabolic diseases	2	2	2
V. Mental and behavioral disorders	2	2	2
VI. Diseases of the nervous system	1	0	1
VII. Diseases of the eye and adnexa	5	2	7
VIII. Diseases of the ear and mastoid process	0	0	0
IX. Diseases of the circulatory system	4	6	6
X. Diseases of the respiratory system	7	6	8
XI. Diseases of the digestive system	4	1	4
XII. Diseases of the skin and subcutaneous tissue	5	2	5
XIII. Diseases of the musculoskeletal system and connective tissue	8	10	11
XIV. Diseases of the genitourinary system	3	1	3
Total, n (%)	53 (80)	40 (61)	66 (100)

^a^*ICD-10*: *International Statistical Classification of Diseases, Tenth Revision*.

^b^Removing apps offered in both platforms but evaluated individually.

**Figure 3:**
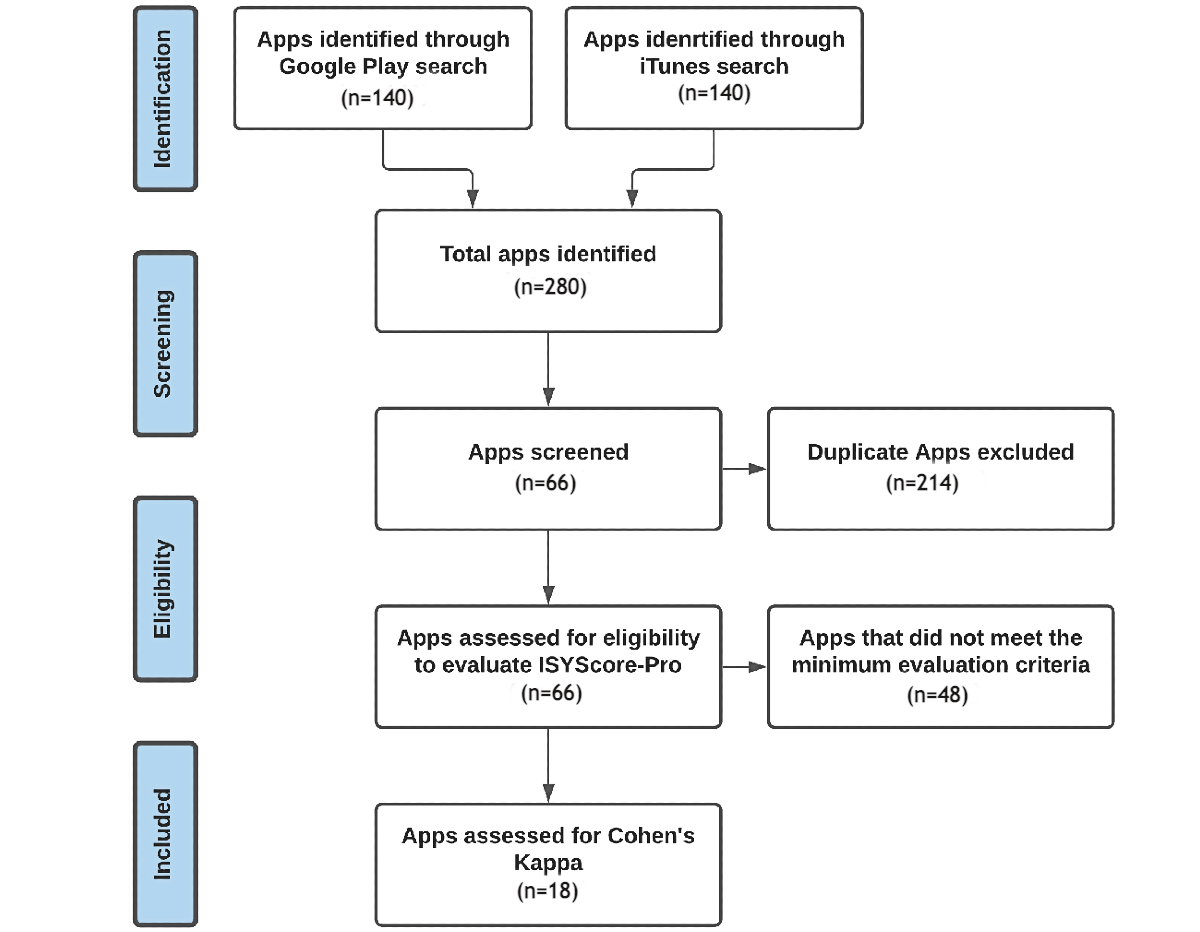
Flow diagram of the app evaluation.

The flow of apps through the research process is shown on Figure 3 using a modified PRISMA (Preferred Reporting Items for Systematic Reviews and Meta‑Analysis) flow diagram.

Results of the evaluation of the agreement between raters, weighted by *ICD-10* group cluster and app, are shown in Table 2. Other analyses are shown in Multimedia Appendix 4. A 92.2% crude general interrater agreement was found, 93.7% and 91.0% when adjusted by cluster and app, respectively. Cohen kappa showed a significantly general agreement (0.84, 95% CI 0.834-0.847), without differences when weighted by app cluster or app evaluated.

**Table 2:** Evaluation of the agreement between raters, weighted by *ICD-10*^a^ group cluster and app.

Evaluation		Agreement (%)	Expected agreement (%)	Cohen kappa (95% CI)	P value
**General (crude)**		92.2	50.8	.84 (0.834-0.847)	<.001
	Weighted by *ICD-10* cluster	93.7	53.1	.87 (0.865-0.867)	<.001
	Weighted by app	91.0	52.5	.81 (0.809-0.810)	<.001

^a^*ICD-10*: *International Statistical Classification of Diseases, Tenth Revision*.

## Discussion

### ISYScore-Pro Tool Development

The 17-item scale ISYScore-Pro is specific to mobile apps targeted to health professionals. ISYScore-Pro assesses apps in the three dimensions that were identified by a literature review [8-10,21]. Other scales in existence consider other dimensions and are often centered upon perception and usability [32-34]. The prioritization of the dimensions on the ISYScore-Pro scale are specific to mobile apps targeted to health care professionals and their practice.

During the Delphi, all dimensions and items on the scale reached strong external panel of experts consensus within two rounds with the exception of C2 (Textbox 1), which was removed from the scale. No further rounds were considered necessary. During the pilot period, an emphasis was placed in selecting health care professionals with different backgrounds across each of the local investigation group team pairs. The piloting also revealed that the scale was easy to apply. The few discrepancies that were encountered in the process were discussed, and consensus was reached in all cases. These results are confirmed by the reported interrater agreement.

Our findings are congruent with previously published research [17,25]. Of particular relevance are the findings of Gagnon and colleagues [25] that found that the main adoption factors of mobile apps by health professionals are perceived usefulness and ease of use, design and technical concerns, cost, time, privacy and security issues, familiarity with the technology, risk-benefit assessment, and interaction with others. The ISYScore-Pro scale addresses a number of these factors including the risk-benefit assessment in the trust domain, perceived utility, and interactions with others (colleagues, patients, and management). The Spanish apps in our sample had low perception of interest, an acceptable level of trust, and an improved perception of utility.

Validating and improving the development of scales specific to mobile apps targeted to health care workers is essential in the future. Ultimately, this will improve the quality, reliability, and usability of these apps, and may improve equity of information whenever and wherever care is delivered.

### Limitations

It would have been difficult to develop the scale using only a systematic review due to the lack of peer-reviewed papers and our current state of knowledge on evaluation tools for smartphone apps targeted to health care professionals. Nevertheless, this issue was overcome with the use of a 5-step framework and a Delphi process.

A limitation of our method was using only volunteers in the external panel members and the investigator group. To mitigate this, a diverse set of professional practice and research experience was used to select the individuals that participated in the Delphi process. Additionally, no conflict of interest was reported by any of the researchers and experts in the study. Another limitation of the ISYScore-Pro scale is that the security, privacy, legal compliance, and efficiency [35] are not assessed by the scale.

It is important to note that it is difficult to find useful apps for health professionals in the Spanish language. As of the time of the study, the market is small, and penetration remains low when compared to the English language. The sample apps that were evaluated had few downloads and even fewer ratings (ie, usually between 500 and 1000).

### Future Research

Although the ISYScore-Pro scale is specific to the Spanish language, future research should compare and contrast our findings with expert opinions in other languages and, in particular, clinicians who practice in the English language. Although our methodology is robust, it is also resource intensive, and the use of automated artificial intelligence and machine learning methods may facilitate and substantially reduce the level of resources needed to assess apps on the market.

The domain of data security in mobile apps was not evaluated. Some authors [7,36] have suggested using open-source developer codes to reduce the potential of malicious functionalities. Future research should evaluate the security, privacy, and integrity of apps and the quality of information contained within them.

### Conclusion

Our research is the first empirical attempt at developing a scale that assesses mobile apps in Spanish targeted to health care professionals. The ISYScore-Pro scale uses a reliable and replicable methodology that standardizes the assessment of trust, utility, and interest using 17 criteria grounded on the existing peer-reviewed literature and the inputs of an expert panel of health care professionals.

## Appendix

Multimedia Appendix 1 Local investigators group (LIG) profiles.

Multimedia Appendix 2 External panel of experts (EPE) profiles.

Multimedia Appendix 3 Strategy in Spanish for each International Statistical Classification of Diseases and Related Health Problems (Tenth Revision; ICD-10) cluster search on Google Play and iTunes.

Multimedia Appendix 4 Evaluation of the agreement between raters, by groups of evaluation, by application, and by item.

## Abbreviations

*ICD-10*: *International Statistical Classification of Diseases, Tenth Revision*
ISYS: Internet, Salud y Sociedad
mHealth: mobile health
PRISMA: Preferred Reporting Items for Systematic Reviews and Meta‑Analysis
