# Supplementary material for: Assessing Apps for Health Care Workers Using the ISYScore-Pro Scale: Development and Validation Study
Source: JMIR Mhealth Uhealth. 2021 Jul 21;9(7):e17660. doi: 10.2196/17660 (PMC8339980; doi:10.2196/17660)
Supplement: Multimedia Appendix 1 [file mhealth_v9i7e17660_app1.docx]

Multimedia Appendix 1. Local Investigators Group profiles

| *Inmaculada Grau* | Research officer, **Engineer**, PhD on Knowledge and Information Society, Head of the mHealth observatory at the Clinic Hospital of Barcelona. Experience in mobile app evaluation. President of the ISYS Foundation |
| --- | --- |
| *Francisco Grajales III* | PhD on Big Data, **Medical Doctor**, Masters on Science, Knowledge Synthesis and Management. Senior researcher with more than eight years on digital investigation |
| *Belchin Kostov* | Research Biostatistician, and more than seven years on Primary Healthcare Transversal Research Group |
| *Valentí Aragunde* | **General Practitioner**, seventeen years on Primary Healthcare Transversal Research Group |
| *Marta Puig* | **General Pract**itioner, ten years on Primary Healthcare Transversal Research Group |
| *Daria Roca* | Nurse, patient educator, specialist on patient communication strategies for therapeutics, ten years on Primary Healthcare Transversal Research Group |
| *P Efrain Pantoja* | PhD candidate, **Medical Doctor**, Masters on Public Health, Epidemiologist, Specialist on Preventive Medicine. Four years’ experience in research on mobile app evaluation. One-year experience on systematic reviews at the Ibero-american Cochrane Network |
| *Elvira Couto* | **General Practitioner**, two years on Primary Healthcare Transversal Research Group |
