# Supplementary material for: Assessing Apps for Health Care Workers Using the ISYScore-Pro Scale: Development and Validation Study
Source: JMIR Mhealth Uhealth. 2021 Jul 21;9(7):e17660. doi: 10.2196/17660 (PMC8339980; doi:10.2196/17660)
Supplement: Multimedia Appendix 2 [file mhealth_v9i7e17660_app2.docx]

Multimedia Appendix 2. External Panel of Experts profiles

| Manuel Escobar | Information and technology, health engineer. Mobile app development |
| --- | --- |
| Luis Fernández Luque | Information and technology, health engineer. Mobile app development |
| Victor Bautista | Information and technology, health engineer. Mobile app development |
| Jose Juan Gomez | Medical doctor, cardiologist, editor of [cardio2cero.com](http://www.cardio2cero.com) |
| Amalia Arce | Medical doctor, paediatrician, editor of [dra-amalia-arce.com](http://www.dra-amalia-arce.com) |
| Rosa Taberner | Medical doctor, dermatologist, editor member of [dermapixel.com](http://www.dermapixel.com) |
| Joan Fontdevila | Medical doctor, surgeon, team member of doctoralia.es |
| Joan Carles March | Medical doctor, public health doctor, head of interoperability of clinical information program at the Clinic Hospital in Barcelona |
| Frederic Llordachs | Medical doctor, entrepreneur, founder of www.doctoralia.es |
| Joan Escarrabill | Medical doctor, pneumologist, senior researcher, Head of Patient Experience at the Barcelona Clinic Hospital |
| Mireia Sans | Medical doctor, family doctor specialist, board-member at the Barcelona School of Medicine of the medical technologies, information and communication board |
| Antoni Benabarre | Medical doctor, psychiatrist, senior researcher at the Barcelona Clinic Hospital |
| Jordi Vilardell | Medical doctor, nephrologist, member at the communication board and senior researcher at the Barcelona Clinic Hospital |
| Anna Sort | Nurse, CEO of @play_benefit, Author of "From Games to Health" |
| Jose María Cepeda | Nurse, creator of saludconectada.com |
| Marga Jansa | Nurse, specialist in diabetes, editor member of socialdiabetes.es |
| Rosa Pérez | Nurse, anthropologist, health educator, creator of elblogderosa.es |
| Marc Fortes | Nurse, anthropologist, health educator, director of infermeravirtual.com/ |
| Lluís Gonzalez | Nurse, researcher at the Barcelona Centre of Primary Health Attention |
| Imma Male | Nurse, coordinator of technology, information and communication group in aificc.cat |
| Jordi Vilaro | Physiotherapist and rehabilitator, specialist in respiratory diseases, clinical researcher |
| Luna Couto Jaime | Sport and physical activities specialist, creator of @luna_couto (Instagram) |
| Javi Telez | Pharmaceutic, Social Media Manager at TEVA Pharmaceuticals Europe |
| Mónica Moro | **Medical doctor**, masters on communication, responsible of ebusiness on Menarini Group Spain |
| Pau Gascón | **Pharmaceutic**, masters on business and administration, digital transformation project management at the Barcelona Clinic Hospital |
| Marisa Ara | **Psychologist**, mental health coordinator on clinical psychology in Hospitalet de Llobregat, Barcelona |
| Manuel Armayones | **Psychologist**, Psychology PhD Health Behavior Design, HIMSS top50 Health IT Leaders Europe. Forbes Spain 2019 Health Best Influencer. Researcher at the UOC eHealth Center |
| Eulalia Hernández | **Psychologist**, PhD Psychology. Associate professor at Universitat Oberta de Catalunya and Researcher at the UOC eHealth Center |
