# Supplementary material for: Assessing Apps for Health Care Workers Using the ISYScore-Pro Scale: Development and Validation Study
Source: JMIR Mhealth Uhealth. 2021 Jul 21;9(7):e17660. doi: 10.2196/17660 (PMC8339980; doi:10.2196/17660)
Supplement: Multimedia Appendix 3 [file mhealth_v9i7e17660_app3.docx]

Multimedia Appendix 3. Strategy in spanish for each ICD-10 cluster search on Google Play and iTunes

| 1 | **Ciertas enfermedades infecciosas y parasitarias** | |
| --- | --- | --- |
|  |  | VIH, Sida, seropositivos, sífilis, hepatitis, virus, vacuna, preservativo, Tuberculosis, Encefalitis, Herpes, Plasmodium, Paludismo |
| 2 | **Neoplasias** | |
|  |  | Linfedema, quimioterapia, radioterapia, tumor, metástasis, linfoma, leucemia, colorectal, ostomía, cáncer, carcinoma, cribaje, mamografía, ganglio centinela, hormonoterapia, inmunoterapia, leucopenia, linfedema, metástasis, mastectomia, neoadyuvante, neoplasia, recidiva, recaída, seroma, tamoxifeno, tumor, tumorectomia, melanoma |
| 3 | **Enfermedades de la sangre y de los órganos hematopoyéticos y otros trastornos que afectan el mecanismo de la inmunidad** | |
|  |  | Coagulopatías, sangrado, coagulación, hematología, hemofilia, Hemorragia, Hemartosis, hemofilia, hemofílico, valvulopatía |
| 4 | **Enfermedades endocrinas, nutricionales y metabólicas** | |
|  |  | Diabetes, diabéticos, insulina, glucosa , periodontitis, Obesidad, hipocalórica |
| 5 | **Trastornos mentales y del comportamiento** | |
|  |  | "Salud mental", alucinaciones, Estigma, "Trastornos Mentales" Esquizofrenia Depresivo, Esquizoafectivo, bipolar |
| 6 | **Enfermedades del sistema nervioso** | |
|  |  | Alzheimer, demencia, cuidadores, esclerosis múltiple Fatiga, brotes, Meningitis, Encefalitis, Encefalomielitis, Ataxia, epilepsia, parkinson |
| 7 | **Enfermedades del ojo y sus anexos** | |
|  |  | Accesibilidad, ceguera, "miopia magna", "baja visión", "miopía patológica", "miopía magna", Ocular, Conjuntivitis, Estrabismo, Glaucoma, Queratitis |
| 8 | **Enfermedades del oído y de la apófisis mastoides** | |
|  |  | Sordo, sordera, lengua de signos, audífonos |
| 9 | **Enfermedades del sistema circulatorio** | |
|  |  | Cardiopatía congénita, Coagulación, coagulómetro, fibrilación auricular, ictus, tromboembólico, Estenosis, Infarto, Trombosis, Varices, Angina, Hipertensiva, Hipotensiva, Miocarditis, Tromboflebitis |
| 10 | **Enfermedades del sistema respiratorio** | |
|  |  | Asma, enfermedades respiratorias, EPOC, enfisema, "bronquitis crónica", alergia, "fibrosis quística", trasplantes, "fisioterapia respiratoria", tabaquismo, "apnea del sueño", "Hipertensión portal" |
| 11 | **Enfermedades del aparato digestivo** | |
|  |  | "Trasplante hepático", "donantes de órganos", HCrohn, "colitis ulcerosa", ostomía, hepatocarcinoma, hepatotoxicidad, "hígado graso", "cirrosis hepática", "Trasplante Vivo", "Atresia biliar" |
| 12 | **Enfermedades de la piel y el tejido subcutáneo** | |
|  |  | Lupus, autoinmune, Dermatitis, Eritema, Urticaria, Psoriasis, Purito |
| 13 | **Enfermedades del sistema osteomuscular y del tejido conectivo** | |
|  |  | Artritis, espondiloartritis, fractura, cadera, vertebra, osteoporosis, artritis reumatoide, articulación, inflamación, reumatología, |
| 14 | **Enfermedades del aparato genitourinario** | |
|  |  | Riñón diálisis "trasplante renal" peritoneal poliquistosis litiasis "donación de órganos" |
