# Supplementary material for: Assessing Apps for Health Care Workers Using the ISYScore-Pro Scale: Development and Validation Study
Source: JMIR Mhealth Uhealth. 2021 Jul 21;9(7):e17660. doi: 10.2196/17660 (PMC8339980; doi:10.2196/17660)
Supplement: Multimedia Appendix 4 [file mhealth_v9i7e17660_app4.docx]

Multimedia Appendix 4. Evaluation of the agreement between raters, by groups of evaluation, by application and, by item.

|  |  | **Agreement** | **Expected Agreement** | **Cohen Kappa (IC95%)** | | **p** |
| --- | --- | --- | --- | --- | --- | --- |
|  |  | **(%)** | **(%)** |  |  |  |
| **By evaluating evaluator pair** | | | | | | |
|  | PEP/EC | 92.2 | 49.9 | 0.84 | (0.824-0.862) | <0.0001 |
|  | MP/IG | 96.6 | 51.3 | 0.93 | (0.915-0.947) | <0.0001 |
|  | DR/VA | 85.9 | 52.0 | 0.71 | (0.683-0.729) | <0.0001 |
|  |  |  |  |  |  |  |
| **By application (n=18)** | | | | | | |
|  | EPIC Diabetes | 82.4 | 52.6 | 0.63 | (0.513-0.742) | 0.0045 |
|  | GenHem | 82.4 | 49.1 | 0.65 | (0.545-.0761) | 0.002 |
|  | Fotoskin | 94.1 | 50.5 | 0.88 | (0.767-0.996) | 0.0001 |
|  | Prevensuic | 94.1 | 50.5 | 0.88 | (0.767-0.996) | 0.0001 |
|  | EPOC Respira | 100.0 | 50.2 | 1.00 | (0.885-1.115) | <0.0001 |
|  | Espiro | 100.0 | 50.2 | 1.00 | (0.885-1.115) | <0.0001 |
|  | SER | 100.0 | 50.2 | 1.00 | (0.885-1.115) | <0.0001 |
|  | 30 años de VIH | 94.1 | 50.5 | 0.88 | (0.767-0.996) | 0.0001 |
|  | Tiempos Ictus | 100.0 | 50.2 | 1.00 | (0.885-1.115) | <0.0001 |
|  | Artroscopia cadera | 100.0 | 50.2 | 1.00 | (0.885-1.115) | <0.0001 |
|  | Workstation en Hematología | 100.0 | 51.6 | 1.00 | (0.885-1.115) | <0.0001 |
|  | Diario del Paciente Ostomizado | 100.0 | 50.2 | 1.00 | (0.885-1.115) | <0.0001 |
|  | PsoriasisCalc | 100.0 | 51.6 | 1.00 | (0.885-1.115) | <0.0001 |
|  | Simposio Sida y Hepatitis 2016 | 82.4 | 50.5 | 0.64 | (0.529-0.758) | 0.0038 |
|  | MetoApp | 100.0 | 54.3 | 1.00 | (0.885-1.115) | <0.0001 |
|  | PDI | 46.1 | 56.1 | 0.20 | (0.090-0.319) | 0.8028 |
|  | FIT Cáncer 2 | 94.1 | 67.1 | 0.82 | (0.708-0.934) | 0.0003 |
|  | Trasplantes 2016 | 88.2 | 64.0 | 0.67 | (0.558-0.788) | 0.0028 |
|  |  |  |  |  |  |  |
| **By item evaluated** | | | | | | |
|  | **A. Trust** |  |  |  |  |  |
|  | A1. Validated by an organization | 88.9 | 51.9 | 0.77 | (0.663-0.875) | 0.0004 |
|  | A2. Identifies the authors | 83.3 | 48.8 | 0.67 | (0.571-0.778) | 0.0012 |
|  | A3. Has a website (responsibility) | 83.3 | 50.0 | 0.67 | (0.564-0.769) | 0.0013 |
|  | A4. Cites sources | 83.3 | 50.0 | 0.67 | (0.564-0.769) | 0.0013 |
|  | A5. Names the organization responsible | 94.4 | 57.4 | 0.87 | (0.762-0.978) | 0.0001 |
|  | A6. Last update less than 1 year ago | 94.4 | NC^a^ | NC^a^ | NC^a^ | NC^a^ |
|  | A7. Discloses how the app is financed | 83.3 | 53.7 | 0.64 | (0.532-0.748) | 0.0031 |
|  | **B. Utility** |  |  |  |  |  |
|  | B1. Provides calculators and measurements | 94.4 | 50.0 | 0.89 | (0.781-0.997) | 0.0001 |
|  | B2. Helps in a care procedure | 83.3 | 57.4 | 0.61 | (0.501-0.717) | 0.0046 |
|  | B3. Archives data images | 94.1 | 49.8 | 0.88 | (0.768-0.997) | 0.0001 |
|  | B4. Facilitates observation of causality | 100.0 | 89.5 | 1.00 | (0.891-1.109) | <0.0001 |
|  | B5. Facilitates vicarious learning | 100.0 | 89.5 | 1.00 | (0.891-1.109) | <0.0001 |
|  | B6. Facilitates patient follow-up | 100.0 | 50.6 | 1.00 | (0.891-1.109) | <0.0001 |
|  | B7 Obtains positive feedback | 100.0 | NA^b^ | NA^b^ | NA^b^ | NA^b^ |
|  | B8. Provides social content | 94.4 | NA^a^ | NA^a^ | NA^a^ | NA^a^ |
|  | **C. Interest** |  |  |  |  |  |
|  | Positive Rating telling users downloads/ user rating | 94.4 | NA^a^ | NA^a^ | NA^a^ | NA^a^ |
|  | Available at least 2 platforms | 100.0 | 64.0 | 1.00 | (0.885-1.115) | <0.0001 |
| NA: Not possible to be calculated, too few discrepancies by both investigators.  ^a^: Most of the categories were rated with the same category.  ^b^: No discrepancies, all categories were rated with the same category. | | | | | | |
